# Supplementary material for: Climate policy uncertainty and its impact on real estate market dynamics: A sectoral and regional analysis
Source: PLoS One. 2024 Oct 25;19(10):e0311688. doi: 10.1371/journal.pone.0311688 (PMC11508155; doi:10.1371/journal.pone.0311688)
Supplement: Attachment — Submitted filename: Response to Reviewers.docx [file pone.0311688.s001.docx]

Manuscript PONE-D-24-24485

Response to Reviewers

Dear Dr. Marco Maria Sorge,

Thank you for giving us the opportunity to submit a revised draft of the manuscript “Climate Policy Uncertainty and Its Impact on Real Estate Market Dynamics: A Sectoral and Regional Analysis” for publication in the PLOS One. We appreciate the time and effort that you and the reviewers dedicated to providing feedback on our manuscript and are grateful for the insightful comments on and valuable improvements to our paper. We have incorporated most of the suggestions made by the reviewers. Those changes are highlighted within the manuscript. Please see below, in blue, for a point-by-point response to the reviewers’ comments and concerns. All page numbers refer to the revised manuscript file with tracked changes. We believe that these modifications have strengthened the manuscript and hope that the revised manuscript is suitable for publication in PLOS One.

Sincerely,

Beakcheol Jang

Graduate School of Information, Yonsei University

03722

+82-10-8516-8355

+82-2-2123-4198

bjang@yonsei.ac.kr

**PONE-D-24-24485 - Reviewer 1**

**Climate Policy Uncertainty and Its Impact on Real Estate Market Dynamics: A Sectoral and Regional Analysis**

Thank you for another opportunity to revise our manuscript. Our revision again benefitted from your and the editor’s writing guidance. We made a sincere effort to address all of the comments we received and, as a result, have made substantial writing changes to the paper. We hope that these changes and clarifications significantly improve the paper and alleviate the concerns you and the reviewer raised about the previous draft. We look forward to your feedback and would, of course, be happy to make further changes.

Below we provide responses to your comments on our paper (reproduced in bold, blue italics).

***Comment 1: There is no evidence of a model selection procedure for choosing the augmented and baseline models. This task should be mandatory, especially in selecting the order of the autoregressive process. Actually, if the model is bad-specified, any further efforts of measuring the predictive ability of CPU may be unhelpful and the Diebold and Mariano test can be misleading. Instead, the authors follow the approach of Paye (2012) without providing a justification for why the AR(1) model is better suited to the data.***

Author Response: Thank you very much for your insightful feedback and for highlighting the importance of a rigorous model selection procedure. We fully agree that ensuring the proper specification of our models is critical for the robustness of our results, particularly when assessing the predictive ability of CPU and conducting the Diebold and Mariano test.

In response to your concern, we conducted a thorough model selection process to determine the optimal order of the autoregressive (AR) process for both the augmented and baseline models. Specifically, we compared the performance of AR(1), AR(2), and AR(3) models using the Akaike Information Criterion (AIC), Bayesian Information Criterion (BIC), and Hannan-Quinn Criterion (HQC) across various specifications.

We’ve added suggested content as below;

**[Line 129 (p.6) ~ Line 138 (p.7)]**

“ *The decision to utilize the AR(1) model was based on a rigorous model selection process. We compared the performance of AR(1), AR(2) and AR(3) models using the Akaike Information Criterion (AIC), Bayesian Information Criterion (BIC), and Hannan-Quinn Criterion (HQC). Table 1. shows the comparison of AIC, BIC and HQC values for AR(1), AR(2) and AR(3). Across all model specifications, the AR(1) model consistently demonstrated the lowest AIC, BIC and HQC values, indicating that it provides the best balance between model fit and complexity. These finding confirm that the AR(1) model is the most appropriate for capturing the dynamics of our dataset, minimizing the risk of overfitting while enabling robust and reliable results.*”

Table 1 Comparison of AIC, BIC, and HQC values for AR(1), AR(2), and AR(3) Models Across Different Real Estate Indices Model

| Model | AR(1) | | | AR(2) | | | AR(3) | | |
| --- | --- | --- | --- | --- | --- | --- | --- | --- | --- |
|  | AIC | BIC | HQC | AIC | BIC | HQC | AIC | BIC | HQC |
| AR-CPU-Comp. | -1423 | -1413 | -1419.37 | -1295 | -1285 | -1291.4 | -1221 | -1211 | -1216.62 |
| AR-CPU-Apart. | -1457 | -1447 | -1453.26 | -1334 | -1324 | -1329.89 | -1275 | -1265 | -1271.09 |
| AR-CPU-Ind. | -1376 | -1366 | -1371.63 | -1212 | -1203 | -1208.44 | -1120 | -1110 | -1116.23 |
| AR-CPU-Ret. | -1304 | -1294 | -1299.81 | -1135 | -1125 | -1131.23 | -1017 | -1008 | -1013.44 |
| AR-CPU-Mall. | -1248 | -1238 | -1243.98 | -1108 | -1098 | -1103.69 | -1017 | -1008 | -1013.47 |
| AR-CPU-F.Outlet | -1325 | -1315 | -1321.17 | -1249 | -1239 | -1244.96 | -1174 | -1164 | -1170.23 |
| AR-CPU-Hotel. | -1269 | -1259 | -1265.17 | -1165 | -1155 | -1160.62 | -1085 | -1075 | -1080.57 |
| AR-CPU-Mfchome. | -1433 | -1423 | -1429.2 | -1281 | -1271 | -1276.89 | -1185 | -1176 | -1181.35 |
| AR-CPU-Office. | -1390 | -1380 | -1385.73 | -1233 | -1223 | -1228.82 | -1129 | -1119 | -1125.25 |
| AR-CPU-Resident. | -1399 | -1389 | -1395.38 | -1220 | -1211 | -1216.52 | -1108 | -1098 | -1103.99 |

As shown in the table, the AR(1) model consistently yields the lowest AIC, BIC, and HQC values across all model specifications. This indicates that the AR(1) model is the most appropriate choice for capturing the dynamics in our dataset. The consistently lower values across these criteria suggest that the AR(1) model effectively balances model fit with complexity, minimizing the risk of overfitting while providing reliable predictive results.

Based on these findings, we are confident that the AR(1) model is not only theoretically sound but also empirically validated as the most suitable model for our analysis. This rigorous model selection process addresses the concerns you raised and ensures that our results are robust and meaningful.

We appreciate your constructive feedback and hope this explanation clarifies the rationale behind our model choice. We are committed to maintaining the highest standards of methodological rigor and believe that our approach aligns with best practices in the field.

***Comment 2: Another point of concern is that the authors only focus on the comparison of the base model with the extended model without showing preliminary results of the univariate regressions. In my opinion, this omission prevents the reader from fully understanding the magnitude and relationship of the estimated beta parameters with the real estate indices.***

Author Response: Thank you very much for your insightful comment. We understand and appreciate the importance of providing univariate regression analyses to offer a clear and direct view of the relationship between the CPU index and real estate indices. We acknowledge that such analyses can be valuable in understanding the individual impact of the CPU index on each real estate index, particularly in terms of estimating the beta parameters.

We believe that univariate regression may not fully capture the complexities involved. Real estate markets often exhibit lagged responses to external factors such as climate policy uncertainty (CPU), meaning that the effects of CPU might not be immediate but could unfold over time. The inherent delay in the market’s response to external shocks is a key characteristic that univariate regression, which does not account for temporal effects, may not adequately address.

We added below to guide readers why we chose the methodology.

**[Line 108 (p.5) ~ Line 111 (p5.)]**

“*We hope this approach addresses your concerns while also maintaining the integrity of the analysis. We deeply appreciate your guidance in refining our work and are open to any further suggestions you might have.*”

In light of this, we have primarily focused on the AR(1) model and its extended versions in our analysis. These models are designed to incorporate lagged variables, allowing for a more accurate depiction of the temporal dynamics at play. By including lagged terms, the AR(1) model captures the delayed impact of CPU on real estate indices, providing a more nuanced understanding of how these effects evolve over time. This approach better reflects the market’s complexity, where multiple factors interact in a time-dependent manner.

***Comment 3: In addition, other controls may be included in the extended and base models to capture other dynamics in the considered real estate indices, such as market risk, interest rate risk, and credit risk.***

Author Response: Thank you for your valuable suggestion. We fully agree that incorporating additional controls, such as market risk, interest rate risk, and credit risk, would provide a more comprehensive understanding of the dynamics influencing real estate indices. We view this as a valuable direction for future research and plan to explore these additional dimensions in subsequent studies. We now emphasize the importance of including these factors in future research to capture the full range of dynamics that impact real estate indices, alongside the broader application of the CPU index across diverse countries and property types.

We’ve added below to clearly express your concern;

**[Line 343 (p.20) ~ Line 344 (p.20)]**

“Additionally, future studies should consider including other controls, such as market risk, interest rate risk, and credit risk, in both the base and extended models to capture the full range of dynamics influencing real estate indices.”

***Comment 4: Furthermore, the “Robustness Check” section provides an analysis of the heteroscedasticity and autocorrelation of residuals. Regarding the former, the authors show that, in general, there is no heteroscedasticity, except for the "industrial model," where they use HC3 to adjust standard errors. Regarding autocorrelation, they perform the Durbin-Watson (DW) test, testing only for 1-lag, with no autocorrelation found. Citing the authors: "Results from the DW test for all models approached a value of 2, indicating an absence of serial correlation." However, nothing is done about higher-order autocorrelation. The Breusch-Godfrey procedure can be performed to test for higher-order residual autocorrelation, and the heteroskedasticity and autocorrelation consistent (HAC) covariance estimator should be considered. Additionally, a graphical approach to residuals inspection may help readers better understand the nature of the results.***

Author Response: **(The revised section is [Line 281 p.17 ~ Line 291 p.18])** We appreciate the reviewer’s suggestion to further investigate higher-order autocorrelation. In response, we conducted the Breusch-Godfrey test to assess the presence of higher-order autocorrelation in the residuals of our models. As shown in Table 7, the test results indicate that most models do not exhibit significant higher-order autocorrelation, with p-values exceeding the conventional significance level of 0.05. However, the F.Outlet and Hotel showed potential higher-order autocorrelation, with p-values of 0.002 and 0.054, respectively.

To address this issue, we applied the HAC (Heteroscedasticity and Autocorrelation Consistent) covariance estimator to these models. The results revealed that the F.Outlet model had t-statistics and p-value 1.052/0.002, while the Hodel model had values of 0.316/0.054. Notably, these findings are consistent with the out-of-sample R^2^ result, which also indicated lower explanatory power for these models. Specifically, the lack of statistical significance for the CPU variable in these models suggests that climate policy uncertainty does not significantly influence the volatility of these particular real estate sectors.

Table 7 Results of Breuch-Godfrey Test for Higher-Order Autocorrelation

|  | LM Statistics | p-value |
| --- | --- | --- |
| AR-CPU-Comp. | 1.06 | 0.78 |
| AR-CPU-Apart. | 2.65 | 0.45 |
| AR-CPU-Ind. | 1.6 | 0.66 |
| AR-CPU-Ret. | 2.24 | 0.523 |
| AR-CPU-Mall. | 2.45 | 0.481 |
| AR-CPU-F.Outlet | 14.7 | 0.002 |
| AR-CPU-Hotel. | 7.89 | 0.054 |
| AR-CPU-Mfchome. | 1.95 | 0.583 |
| AR-CPU-Office. | 2.04 | 0.564 |
| AR-CPU-Resident. | 2.41 | 0.492 |

We believe that these additional analyses enhance the robustness of our findings and address the concerns raised regarding higher-order autocorrelation.

**PONE-D-24-24485 - Reviewer 2**

**Climate Policy Uncertainty and Its Impact on Real Estate Market Dynamics: A Sectoral and Regional Analysis**

Thank you for another opportunity to revise our manuscript. Our revision again benefitted from your and the editor’s writing guidance. We made a sincere effort to address all of the comments we received and, as a result, have made substantial writing changes to the paper. We hope that these changes and clarifications significantly improve the paper and alleviate the concerns you and the reviewer raised about the previous draft. We look forward to your feedback and would, of course, be happy to make further changes.

Below we provide responses to your comments on our paper (reproduced in bold, blue italics).

***Comment 1: The topic and analysis is robust. Literature review can be enhanced. Examples below. 1.Maurizio d’Amato, Asma Salman, Giulia Mastrodonato and Giampiero Sirleo. (September 2022) Measures of Variability in the Application of Cyclical Capitalization (normal form) of the London Market. Springer Book chapter from the Book Property Valuation and Market Cycle. Book Chapter indexed in Scopus and Clarivate Analytics***

***https://link.springer.com/book/10.1007/978-3-031-09450-7***

Author Response: Thank you for your positive feedback on the topic and analysis. We appreciate your suggestion to enhance the literature review by including more recent and relevant sources. Following your recommendation, we have incorporated the work of Maurizio et al. (2022) into our literature review. This addition strengthens our discussion on the cyclical nature of the real estate market and its implications for investment strategies and market dynamics.

Specifically, we have revised the literature review to include the following passage:

**[Line 38 (p.3) ~ Line 43 (p.3)]**

"*While the real estate market is influenced by a variety of factors, including economic cycles, market dynamics, and regulatory changes, the impact of climate policy uncertainty introduces an additional layer of complexity. The real estate market is already known for its cyclical nature, with fluctuations in property values, transaction volumes, and investment strategies that are driven by both intrinsic factors (such as supply and demand dynamics) and extrinsic factors (such as broader economic trends and policy changes) (Phyrr et al., 1990; Roulac, 2020). These cycles can create significant risks for investors, particularly when combined with the uncertainties introduced by evolving climate policies*."

We believe that this enhancement provides a more comprehensive background for our study and aligns well with your suggestions. Thank you once again for your valuable input, which has helped to improve the overall quality and depth of our literature review.

***Comment 2: The conclusion should have a policy recommendation.***

Author Response: We think this is an excellent suggestion. We have added a policy recommendation.

**[Line 333 (p.19) ~ Line 338 (p.20)]**

“In light of the findings from this study, policymakers should consider integrating the CPU index into the regulatory framework for real estate markets. This could involve establishing guidelines that require real estate developers and investors to account for CPU-related risks in their financial and operational planning. Additionally, policies aimed at mitigating the impact of CPU, such as incentives for energy efficiency upgrades in vulnerable property types like industrial assets, should be prioritized. By doing so, policymakers can help reduce the volatility induced by CPU and promote more stable and sustainable real estate markets, particularly in regions that are most susceptible to climate change.”
